# Identify risk factors affecting participation of Turkish women in mammography screening for breast cancer prevention

**DOI:** 10.1007/s10549-024-07296-9

**Published:** 2024-03-07

**Authors:** Esra Bayrakçeken, Süheyla Yaralı, Ömer Alkan

**Affiliations:** 1 Department of Medical Services and Techniques, Vocational School of Health Services, Ataturk University, Yakutiye/Erzurum, Türkiye; 2 Department of Public Health Nursing, Faculty of Nursing, Ataturk University, 2nd Floor, No:49, Yakutiye/Erzurum, Türkiye; 3 Department of Econometrics, Faculty of Economics and Administrative Sciences, Ataturk University, 2nd Floor, No:222, Yakutiye/Erzurum, Türkiye; 4 Master Araştırma Eğitim ve Danışmanlık Hizmetleri Ltd. şti., Ata Teknokent, Erzurum, TR-25240 Türkiye

**Keywords:** Mammography screening, Cancer, Women’s health, Stereotype logistic regression, Türkiye

## Abstract

**Purpose:**

Cancer screening is a public health intervention aiming to reduce cancer-caused deaths. This study aims to determine the factors affecting the mammography screening time among women aged 40–69.

**Methods:**

The micro dataset obtained from the Türkiye Health Survey conducted by the Turkish Statistical Institute (TurkStat) in 2019 and 2022 was used in the present study. Stereotype logistic regression was used to determine the variables affecting mammography screening and period for breast cancer prevention in women in Türkiye.

**Results:**

Given the results achieved from the analysis, it was found that factors such as age, marital status, general health condition, comorbidity, receiving psychosocial support, high blood lipid levels, and performing breast self-examinations affected women’s adherence to cancer screening programs.

**Conclusion:**

Since adherence to mammography increases with age, it is recommended to pay importance to education for women approaching the age of mammography screening. Educated individuals are expected to have access to multiple sources of information as to cancer and to access this information more easily. In order to gain more insight into the recommended preventive measures and outcomes related to cancer, it is suggested to review policies, which will increase the educational level of women, and provide privileges in the field of education.

**Supplementary Information:**

The online version contains supplementary material available at 10.1007/s10549-024-07296-9.

## Introduction

Cancer is the second leading cause of death worldwide [1]. The top three cancers among individuals over the age of 15 globally are breast, lung, and colon, respectively. The top three cancers among women are breast, colon, lung, and cervical cancer [2]. Breast cancer is a global health problem due to its high prevalence worldwide [3]. It is a heterogeneous disease affected by both genetic and environmental factors [4]. It was reported that menarche at early age, parity, and advanced age at first full-term pregnancy might affect breast cancer risk through long-term effects on hormone levels or other biological mechanisms [5].

There have been 685,000 deaths worldwide from breast cancer [1]. It is reported that 3,311,765 women in Türkiye, approximately 24%, have been diagnosed with breast cancer [2]. Cancer screening is a public health intervention aiming to reduce cancer-related deaths [6]. Early detection of cancer is crucial for cancer control. Screening and early detection programs are implemented in order to ensure effective cancer treatment [7]. Breast cancer screening programs are systems aiming to reduce breast cancer-related deaths by means of early detection and effective treatment [8].

In the literature, initiation of breast cancer screening at the ages of 40–41 years or 50 years was studied and it was determined that reducing the starting age from 50 years to 40 years could potentially reduce breast cancer-related deaths [9]. It was reported that participation in a breast cancer screening program reduces the death rate by 40% among women aged between 40 and 74 years [10]. In a cross-sectional study carried out in a province in Türkiye, it was determined that 8,758 women aged between 40 and 69 years were screened every two years with bilateral mammography, and 130 women were diagnosed with breast cancer during a 10-year screening period [11]. A study carried out in the USA revealed that, in Michigan, 40.4% of Arab women in Michigan had clinical breast examinations and mammograms in the past year, when compared to 50.4% of all women aged 40 years and older [12]. In Türkiye, women aged between 40 and 69 years are recommended to have a mammogram every two years as part of a breast cancer screening program [13]. Mammography screening can prevent approximately 25% of deaths caused by breast cancer [14]. It is estimated that, if all countries achieve 100% examination coverage, then 12,434 breast cancer deaths could be prevented every year [15].

Participation in early cancer diagnosis programs is affected by many factors. Identifying the effective factors is important in order to ensure women’s participation in cancer screening programs. Therefore, this study aims to answer the question “What are the factors playing an effective role in the participation of women in the breast cancer screening program in Türkiye?” For this purpose, factors influencing women’s participation in early diagnosis programs were modeled for Türkiye by using a rich dataset.

## Method

### Data

It was reported that the community-based and standardized mammography screening can reduce breast cancer deaths by 25% in the age group of 50–69 years and by 40% in the age group of 40–69 years. Mammography screening is performed every two years for women aged between 40 and 69 years by following the National Standards of the Breast Cancer Screening Program in order to achieve early diagnosis in Türkiye [16].

Türkiye Health survey was realized to provide indicators in health areas not obtained by the administrative registration system and constitute a data source for the decision-makers and researchers. Türkiye Health Survey aims to get information about health indicators, which include a big part of the development indicators that show the degree of development of the countries. This survey is essential to be the first study that reflects the country as a whole and international and national needs, allowing comparisons in terms of a study that sheds light [17].

Türkiye Health Interview Survey was carried out by TurkStat in 2008 for the first time, and then it was conducted periodically every two years until 2016. Health Survey derives many indicators on health, including health conditions of infants, children, and adults, health services utilisation, difficulties faced during daily activities, and cigarette and alcohol use habits for individuals 15 years old and over. All the individuals living in Türkiye were covered. The institutional population (soldiers, individuals living in dormitories, prisons, hospitals in the long-terms, homes for the elderly, etc.) and the small settlements where a sufficient number of sample households cannot be reached (Small villages, hamlets, etc.) are excluded [17].

A stratified two-step cluster sampling method is used. As the criterion of external stratification, the rural-urban separation is used. (Settlements with a population of 20,000 or less and settlements with a population of 20,001 or above were considered urban). The first stage sampling unit consists of blocks randomly selected with the selection proportional to the size of the clusters (blocks) containing an average of 100 addresses. The second stage sampling unit systematically randomly selected household addresses from each selected cluster [17].

**Fig. 1:**
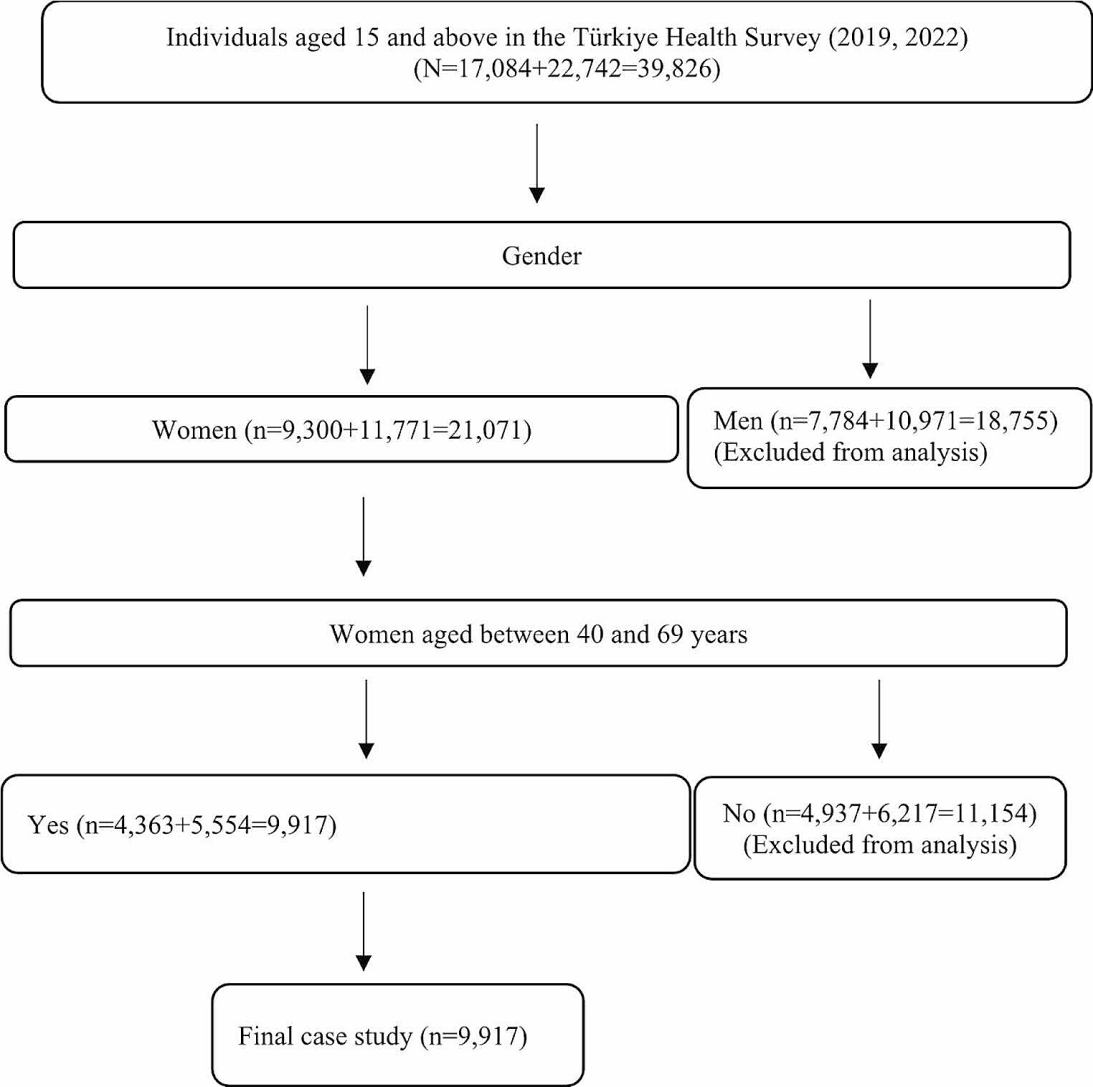
Selection process of women aged between 40 and 69 years among individuals in the THS

The data set from the 2019 and 2022 Health Survey conducted by the Turkish Statistical Institute (TurkStat) was used in the present study. The most recent Türkiye Health Survey data that TurkStat shared is that of 2022. The 2019 Health Survey was conducted with 9,470 households. The 2022 Health Survey was conducted with 11,170 households. In this study, the data obtained from 9,917 women aged between 40 and 69 years in the Türkiye Health Survey for 2019 and 2022 were included in the analysis. The selection process of the sample to be included in the study is given in Fig. 1.

### Outcome variables

The dependent variable in the present study is the time women had their last mammography, measured by the question “When did you last have a mammogram/breast x-ray?”. The dependent variable in the model consists of four categories: “never, less than 2 years ago, more than 2 years and less than 5 years, more than 5 years ago.”

### Independent variables

The independent variables in this study are those found in the Türkiye Health Survey. The independent variables of the study include age (between the ages of 40 and 49 years, between the ages of 50 and 59 years, and between the ages of 60 and 69 years) [13, 18], education (illiterate or did not graduate any school, and elementary school, secondary school, high school, and university graduates) [19–21], marital status (never married, married, and divorced/widowed) [22], general health status (very good/good, moderate, and poor/very poor) [23], having an illness lasted/expected to last longer than 6 months (yes, no) [24], high level of blood lipid [25, 26], receiving psychosocial support (yes, no) [27, 28], and frequency of breast self-examination (monthly, every three months, longer than three months, and never) [29]. Ordinal and nominal variables were defined as dummy variables in order to observe the effects of all categories that will be included in the binary logistic regression model [30].

### Analysis method

Survey statistics in Stata 15 (Stata Corporation) were used to account for the complex sampling design and weights. Weighted analysis was performed. The frequency and percentages of the mammography screening times of the participants and the independent variables were obtained first. The stereotype logistic regression method was used in this study in order to investigate the factors related to mammography screening time for women aged between 40 and 69 years.

## Results

### Descriptive statistics and crosstabs

Descriptive statistics and Chi-Square independence test results for the variables used in this study are presented in Table 1. In 2019, the prevalence of breast cancer screening among women aged 40–69 years participating in the survey was 55.0%, and in 2022, it was 53.0%.

**Table 1:** Findings for the factors influencing the mammography screening time of women

Variables	*n* (%)	Never	Less than 2 years	More than 2 years but less than 5 years	More than 5 years	*P*
**Age**						
40–49	4,037(40.7)	2,128(49.3)	1,100(40.9)	517(31.2)	292(25.1)	0.000^a^
50–59	3,319(33.5)	1,276(28.9)	949(35.3)	657(39.6)	437(37.6)	
60–69	2,561(25.8)	1,005(22.8)	639(23.8)	484(29.2)	433(37.3)	
**Educational Level**						
Illiterate	1,971(19.9)	1,083(4.6)	401(14.9)	285(17.2)	202(17.4)	0.000^a^
Elementary School	4,861 (49)	2,119(48.1)	1,290(48.0)	850(51.3)	602(51.8)	
Secondary School	672(6.8)	288(6.5)	206(7.7)	109(6.6)	69(5.9)	
High School	1,314(13.2)	523(11.9)	398(14.8)	231(13.9)	162(13.9)	
University	1,099(11.1)	396(9.0)	393(14.6)	183(11.0)	127(10.9)	
**Marital Status**						
Not Married	362(3.7)	233(5.3)	65(2.4)	42(2.5)	22(1.9)	0.000^a^
Married	7,884(79.5)	3,483(79.0)	2,174(80.9)	1,334(80.5)	893(76.9)	
Divorced/Widowed	1,671(16.8)	693(15.7)	449(16.7)	282(17.0)	247(21.3)	
**General Health Status**						
Very good/Good	4,092(41.3)	1,992(45.2)	992(36.9)	663(40.0)	445(38.3)	0.002^a^
Moderate	4,415(44.5)	1,829(41.5)	1,291(48.0)	754(45.5)	541(46.6)	
Poor/Very poor	1,410(14.2)	588(13.3)	405(15.1)	241(14.5)	176(15.1)	
**Illness lasting/expected to last longer than 6 months**						
Yes	7,699(77.6)	3,208(72.8)	2,231(83.0)	1,330(80.2)	930(80.0)	0.000^a^
No	2,218(22.4)	1,201(27.2)	457(17.0)	328(19.8)	232(20.0)	
**High level of blood lipids (high cholesterol or triglyceride)**						
Yes	1,869(18.8)	625(14.2)	655(24.4)	363(21.9)	226(19.4)	0.000^a^
No	8,048(81.2)	3,784(85.8)	2,033(75.6)	1,295(78.1)	936(80.6)	
**Receiving psychosocial support**						
Yes	837(8.4)	292(6.6)	314(11.7)	142(8.6)	89(7.7)	0.000^a^
No	9,080(91.6)	4,117(93.4)	2,374(88.3)	1,516(91.4)	1073(92.3)	
**Frequency of Breast Self-Examination**						
Every month	2,593(26.1)	792(18.0)	1,036(38.5)	448(27.0)	317(27.3)	0.000^a^
Every three months	1,018(10.3)	307(7.0)	382(14.2)	216(13.0)	113(9.7)	
Longer than three months	2,030(20.5)	529(12.0)	646(24.0)	520(31.4)	335(28.8)	
Never	4,276(43.1)	2,781(63.1)	624(23.2)	474(28.6)	397(34.2)	

^a^*p*<0.01

Examining the Chi-Square independence test results presented in Table 1, it was determined that all the variables in Table 1 are related with mammography screening time of women.

### Model estimation

It was also examined whether there was multicollinearity among the independent variables in the model. It is considered that variables with Variance Inflation Factor (VIF) values of 5 and above cause moderate multicollinearity, and those with 10 and above cause high multicollinearity [31]. Given the VIF results presented in Table 2, there is no variable causing a multicollinearity issue.

**Table 2:** The coefficients of the stereotype logistic regression model investigating determinants of mammography screening

Variables	β	Std. Err.	95%CI		VIF
			Lower	Upper	
**Age (Ref: 40–49)**					
50–59	0.494^a^	0.066	0.364	0.623	1.280
60–69	0.561^a^	0.083	0.399	0.722	1.430
**Educational Level (Ref: Illiterate)**					
Elementary School	0.396^a^	0.066	0.267	0.525	1.910
Secondary School	0.390^a^	0.102	0.189	0.591	1.320
High School	0.629^a^	0.089	0.454	0.804	1.620
University	0.840^a^	0.098	0.648	1.032	1.600
**Marital Status (Ref: Divorced/Widowed)**					
Not Married	-0.766^a^	0.132	-1.025	-0.508	1.210
Married	0.021	0.059	-0.095	0.138	1.230
**General Health Status (Ref: Very good/Good)**					
Moderate	0.145^a^	0.056	0.034	0.255	1.570
Poor/Very poor	0.195^a^	0.078	0.042	0.401	1.510
**Illness lasting/expected to last longer than 6 months (Ref: No)**					
Yes	0.279^a^	0.063	0.156	0.401	1.410
**High level of blood lipids (high cholesterol or triglyceride) (Ref: No)**					
Yes	0.342^a^	0.061	0.221	0.462	1.120
**Receiving psychosocial support (Ref: No)**					
Yes	0.374^a^	0.087	0.205	0.544	1.030
**Frequency of Breast Self-Examination (Ref: Never)**					
Every month	1.336^a^	0.076	1.187	1.486	1.240
Every three months	1.403^a^	0.094	1.220	1.587	1.140
Longer than three months	1.519^a^	0.097	1.328	1.709	1.200
**phil_1**	1	.	.	.	.
**phil_2**	0	base outcome			
**theta_1**	3.222^a^	0.139	2.948	3.495	
**theta_2**	0	base outcome			

^a^*p*<0.01; ^b^*p*<0.05; ^c^*p*<0.10

Examining Table 2, it was determined that age, educational level, general health status, having a disease, high blood lipid level, receiving psychosocial support, and the frequency of breast self-examination affected the time women get mammography screening.

**Table 3:** Marginal effects of factors influencing the mammography screening time of women

Variables	Less than 2 years		More than 2 years but less than 5 years		More than 5 years	
	dy/dx	Std. Err.	dy/dx	Std. Err.	dy/dx	Std. Err.
**Age (Ref: 40–49)**						
50–59	0.064^a^	0.007	0.031^a^	0.005	0.018^a^	0.003
60–69	0.072^a^	0.008	0.035^a^	0.005	0.020^a^	0.004
**Educational Level (Ref: Illiterate)**						
Elementary School	0.051^a^	0.008	0.026^a^	0.004	0.015^a^	0.003
Secondary School	0.050^a^	0.013	0.025^a^	0.007	0.015^a^	0.004
High School	0.081^a^	0.011	0.040^a^	0.006	0.023^a^	0.004
University	0.109^a^	0.012	0.052^a^	0.007	0.030^a^	0.005
**Marital Status (Ref: Divorced/Widowed)**						
Not Married	-0.094^a^	0.015	-0.049^a^	0.008	-0.030^a^	0.006
Married	0.003	0.008	0.001	0.004	0.001	0.002
**General Health Status (Ref: Very good/Good)**						
Moderate	0.019^a^	0.007	0.009^a^	0.004	0.005^a^	0.002
Poor/Very poor	0.025^a^	0.010	0.012^a^	0.005	0.007^a^	0.003
**Illness lasting/expected to last longer than 6 months (Ref: No)**						
Yes	0.036^a^	0.008	0.018^a^	0.004	0.010^a^	0.003
**High level of blood lipids (high cholesterol or triglyceride) (Ref: No)**						
Yes	0.044^a^	0.009	0.021^a^	0.004	0.010^a^	0.002
**Receiving psychosocial support (Ref: No)**						
Yes	0.048^a^	0.012	0.023^a^	0.005	0.013^a^	0.003
**Frequency of Breast Self-Examination (Ref: Never)**						
Every month	0.187^a^	0.011	0.095^a^	0.006	0.057^a^	0.005
Every three months	0.196^a^	0.013	0.099^a^	0.008	0.059^a^	0.006
Longer than three months	0.212^a^	0.010	0.105^a^	0.008	0.062^a^	0.007

^a^*p*<0.01; ^b^*p*<0.05; ^c^*p*<0.10

Examining Table 3, it can be seen that the likelihood of women aged between 50 and 59 years and between 60 and 69 years to have mammograms taken in less than 2 years is higher by 6.4% and 7.2%, respectively, when compared to those aged between 40 and 49 years. The probability of women aged between 50 and 59 years and between 60 and 69 years having mammograms taken within a 2–5 year time frame is higher by 3.1% and 3.5%, respectively, when compared to those aged between 40 and 49 years. The likelihood of women aged 50–59 and 60–69 to have mammograms taken in more than 5 years compared to those aged between 40 and 49 years is higher by 1.8% and 2.0%, respectively.

Educational level was also found to be a variable influencing the mammography scanning time. The likelihood of women having primary school, elementary school, high school, and university education to have mammograms taken in less than 2 years when compared to illiterate women is higher by 5.1%, 5.0%, 8.1%, and 10.9%, respectively. The probability of women having elementary school, secondary school, high school, and university graduation to have mammograms taken within 2–5 years in comparison to illiterate women is higher by 2.6%, 2.5%, 4.0%, and 5.2%, respectively. The likelihood of women having elementary school, secondary school, high school, and university education to have mammograms taken in more than 5 years when compared to illiterate women is higher by 1.5%, 1.5%, 2.3%, and 3.0%, respectively.

Women, who have never been married, have a 9.4% lower probability of having mammograms taken in less than 2 years when compared to widow or divorcee women. The likelihood of non-married women to have mammograms taken within a 2–5 year-time frame is 4.9% lower than that of widow and divorcee women. The probability of non-married women to have mammograms taken in more than 5 years is 3.0% lower.

It was found that women who rated their general health status as moderate and poor/very poor were 1.9% and 2.5% more likely to have mammography less than 2 years, respectively, compared to women who rated their general health status as very good/good. Women who rated their general health status as moderate and poor/very poor were found to be 0.9% and 1.2% more likely to have mammography in the 2-5-year interval than women with very good or good general health, respectively. Women who rated their general health status as moderate and poor/very poor were found to be 0.5% and 0.7% more likely to have mammography for more than 5 years, respectively.

Women, who have had a health issue for longer than six months, have a 3.6% higher likelihood of having mammograms taken in less than 2 years when compared to women having no health issue. When compared to the women having no health issue for longer than 6 months, the probability of having mammograms taken within a 2-5-year time frame for women having a health problem for more than 6 months is 1.8% higher, and the likelihood of having mammogram in longer than 5 years is 1.0% higher.

Women with high blood lipid problems within the last 12 months are 4.4% more likely to have had a mammogram within less than 2 years when compared to women without high blood lipid problems. Women with high blood lipid problems within the last 12 months were found to be 2.1% more likely to have a mammogram within a time frame of 2–5 years in comparison to those without. Furthermore, women with high blood lipid issues within the past 12 months are 1.0% more likely to have had a mammogram in a period longer than 5 years when compared to women without such issues.

Women who received psychosocial support in the past 12 months have a 4.8% higher likelihood of having mammograms taken in less than 2 years when compared to those, who did not receive such support. The probability of having mammograms taken within a 2-5-year time frame is 2.3% higher among women, who received psychosocial support in the past 12 months, in comparison to women who received no psychosocial support in the past 12 months. The probability of having mammograms taken and for more than 5 years is 1.3% higher among women, who received psychosocial support in the past 12 months, in comparison to women who did not.

Women, who examine their breasts every month, every three months, or more than every three months themselves, have a 18.7%, 19.6%, and 21.2%, respectively, higher likelihood of having mammograms taken in less than 2 years when compared to women, who never do self-examinations. Women, who examine their breasts every month, every three months, or more than every three months themselves, have a 9.5%, 9.9%, and 10.5%, respectively, higher probability of having mammograms taken within a 2–5 year and 5.7%, 5.9%, and 6.2%, respectively, higher probability of having mammogram taken in a period of time longer than 5 years when compared to women, who never do self-examinations.

## Discussion

In this study, it was aimed to investigate the factors influencing the intervals, at which women aged between 40 and 69 years undergo mammography scanning in Türkiye. Given the results achieved in this study, it was determined that the intervals at which women undergo mammography vary by age, educational status, marital status, perceived general health, the presence of health problems, high blood lipid levels, receiving psychosocial support, and frequency of breast self-examinations.

The present study revealed that the age affected the timing preference for mammography among the women in the study group. Women aged between 50 and 59 years and those aged between 60 and 69 years pay more attention to the intervals, at which they undergo mammography, when compared to women aged between 40 and 49 years. In particular, the likelihood of women aged between 50 and 59 years and between 60 and 69 years to have mammography more frequently than every 2 years is higher when compared to women aged between 40 and 49 years. The likelihood for the same group to have mammography intervals between 2 and 5 years and longer than 5 years is also higher than that of women aged between 40 and 49 years. Literature is evaluated a breast cancer risk assessment should be performed for all women starting at age 18. This should include a review of personal and family medical history, as well as a review of modifiable risk factors. All women should receive mammography screening by age 50. Higher-risk women may need to begin screening as early as age [32]. The ACR recommends annual screening beginning at age 40 for women of average risk and earlier and more intensive screening for women at higher-than-average risk [33]. Although there are many breast imaging modalities, mammography is the predominant tool for breast cancer screening. The introduction of digital breast tomosynthesis to mammography has increased cancer detection rates and decreased recall rates. In average-risk women, starting annual screening mammography at the age of 40 years has demonstrated the highest mortality reduction [34]. The National Comprehensive Cancer Network (NCCN), one of the leading cancer research centers, recommends annual mammography starting from age of 40 years. They also suggest mammography every year or every two years between the ages of 40 and 49 years, and annually thereafter [18]. In Türkiye, it is recommended for women aged between 40 and 69 years to undergo mammography every two years [13].

The results achieved in the present study revealed that the high educational status of the women increased the likelihood of adherence to mammography intervals. The literature also indicates that education increases the likelihood of participating in mammography screenings [19–21]. Women may avoid mammography screening due to a lack of information about breast cancer risks [21]. Moreover, it is also believed that reproductive factors that may be related to breast cancer, such as never having given birth and age at first birth and not having given birth before, are more common among highly educated women and that women, who perceive themselves at risk, are also more inclined to undergo mammography screening [21].

The adherence to mammography scanning intervals among single women in the present study was found to be lower in comparison to unmarried participants. Married women are more likely to participate in breast cancer screening programs than unmarried women [35, 36]. A higher likelihood of not undergoing mammography was reported for single women in the literature [22]. Married women are thought to have more positive attitudes towards cancer screening programs [37, 38]. The positive attitudes of women may have caused them to pay more attention to cancer screening program times. It is thought that married people have a more comprehensive social network, which helps them apply for preventive testing [39].

In this study, women perceiving their health status to be ‘moderate’ were found to pay more attention to cancer screening programs than those, who rated their health status as ‘very good.’ Fear of health impairment was stated to play a significant role in decision-making and behavior [23].

Comorbidity increases the participation to cancer screening programs among women in the study group. This is similar to the literature [39]. It was reported that breast cancer mortality is higher among women having chronic diseases [24]. While chronic diseases increase cancer mortality [24], some can also cause cancer [40]. This may have increased the participation of women with comorbidities in cancer screening programs.

Women with high blood lipid levels pay more attention to the timing of cancer screening programs. This might be due to the age-related increase in lipid values and the increase in hospital admissions because of cardiovascular risk [25]. At the same time, due to the diversity of their biological roles, lipids contribute to various aspects of tumor biology such as growth, energy, and redox homeostasis, and also to the spread of cancer cells to develop distant metastases [26]. Healthy women are less likely to be admitted to the hospital, making them less likely to learn about the advantages of mammography. Women with chronic diseases go to the hospital more often for treatment. This suggests that it effectively increases the rate of participation in mammography by women with high blood lipid levels [41].

Women receiving psychosocial support pay more attention to the timing of cancer screening programs. Given the literature, it was determined that individuals having severe mental illnesses such as schizophrenia and bipolar disorder are less likely to participate in cancer screening [27]. The probability of women with schizophrenia and other psychotic diseases to participate in mammography screening is about half that of women not having these diseases [28]. It was reported that efforts should be made in order to increase the participation of women having mental illness in cancer screening. Reported results indicate that educational programs implemented for individuals having severe mental illnesses are associated with higher cervical and breast cancer screening rates, but more effective methods should be used [42]. A previous study noted that women diagnosed with depression and seeing family doctors more frequently are more likely to get screened for cervical cancer [43]. Therefore, it can be concluded that receiving psychosocial support contributes to participation in the cancer screening program.

Women, who perform breast self-examinations, pay more attention to the timing of cancer screening programs when compared to those, who don’t. Breast self-examination plays an important role in the early diagnosis of cancer [29]. It is reported in the literature that most breast cancers are identified by patients [44]. The benefits of breast self-examination may encourage women to perform breast examinations [45].

## Conclusion

In this study, it was determined that age, marital status, overall health, comorbidity, receiving psychosocial support, high lipid levels in blood, and practicing breast self-examination play a significant role in women’s adherence to cancer screening programs. Since adherence to mammography increases with age, it is recommended to pay importance to educational programs addressing women approaching mammography screening age.

In conclusion, identifying the effective factors playing a role in women’s participation in cancer screening programs holds significant importance in enhancing participation and incentives in these programs. These determinant factors should be paid attention in educations aiming to increase participation and incentives in cancer screening programs.

### Electronic supplementary material

Below is the link to the electronic supplementary material.

Supplementary Material 1
